# Metabolic factors accelerate colorectal adenoma recurrence

**DOI:** 10.1186/1471-230X-14-187

**Published:** 2014-10-23

**Authors:** Leo Taniguchi, Takuma Higurashi, Takashi Uchiyama, Yoshinobu Kondo, Eri Uchida, Shiori Uchiyama, Fumitake Jono, Jun Hamanaka, Hitoshi Kuriyama, Yasuo Hata, Hiroki Endo, Hirokazu Takahashi, Hajime Nagase, Nobuyuki Matsuhashi, Atsushi Nakajima

**Affiliations:** Department of Gastroenterology, Chigasaki Municipal Hospital, Chigasaki, Japan; Division of Gastroenterology, Yokohama City University School of Medicine, 3-9 Fukuura, Kanazawa-ku, 236-0004 Yokohama, Kanagawa, Japan; Department of Endocrinology and Metabolism, Chigasaki Municipal Hospital, Chigasaki, Japan; Department of Gastroenterology, Yokohama Rousai Hospital, Yokohama, Japan; Department of Gastroenterology, Tokyo Metropolitan Hiroo Hospital, Tokyo, Japan; Department of Gastroenterology, Machida Municipal Hospital, Machida, Japan; Department of Gastroenterology, Kanto Medical Center, NTT EC, Tokyo, Japan

**Keywords:** Metabolic factor, Colorectal adenoma recurrence

## Abstract

**Background:**

Metabolic factors have been reported to increase the prevalence of colorectal adenomas, however, whether metabolic factors might also accelerate the recurrence after removal of adenomas has not yet been discussed. In this retrospective multicenter study, we clarified the risk factors for adenoma recurrence focusing on metabolic factors.

**Methods:**

We analyzed the medical records of 43,195 patients who had undergone colonoscopy between January 2005 and December 2011 at 5 hospitals in Japan. Of these, the data of 1111 patients who had undergone removal of adenomas at the first screening colonoscopy, and then been followed up by colonoscopy 1 year and 2 years later were analyzed.

**Results:**

The following 8 factors were demonstrated with a multivariate analysis as being associated with colorectal adenomas recurrence: for adenoma-related factors, 5 factors (villous features, grade of dysplasia, location and size of the largest removed adenoma, and number of the removed adenomas) were identified; for metabolic factors and other factors, 3 factors (age, body mass index (BMI), and fasting blood glucose (FBG)) were identified. A risk score (0–10 points) was developed based on these 8 factors. The risk of adenoma recurrence increased as the risk score increased. When the risk score was ≥3 (3–10) points, the odds ratio relative to <3 (0–2) points was 7.07 (95% CIs 5.30–9.43).

**Conclusions:**

In addition to adenoma-related factors (villous features, grade of dysplasia, location, size and number), 3 factors (age, BMI and FBG) were demonstrated to influence the recurrence rate of colorectal adenoma. When the risk score was ≥3, the risk of recurrence was significantly elevated.

## Background

Despite the recent advances in therapeutic modalities for colorectal cancer (CRC), it remains a major cause of mortality worldwide [1]. In most cases, CRC develops through the adenoma–carcinoma sequence, which serves as the rationale for screening and prevention of CRC by colonoscopic examinations. Colonoscopy with removal of adenomas is a powerful tool to reduce the mortality of CRC [1–3]. Surveillance colonoscopy is also recommended after the initial endoscopic removal of adenoma, because of the possibility of development of new tumors [2, 3].

In Japan, no guidelines have been established yet for surveillance colonoscopies. However, in the United States, the American Gastroenterological Association (AGA) guidelines are adopted as the basic protocols for colonoscopic surveillance after initial screening/removal of adenomas. These guidelines recommend stratification of patients at the time of the initial colonoscopy into groups at a low and high risk of subsequent development of more advanced tumors [2]. Patients who have advanced adenomas or multiple (3 or more) adenomas are classified into the high-risk group. Advanced adenomas are defined as adenomas that show high-grade dysplasia or >20% villous component or measure ≥1 cm in size. Follow-up colonoscopy every 3 years is recommended for the high-risk group. Patients with other adenomas, namely, 1 or 2 small (<1 cm) tubular adenomas that do not show high-grade dysplasia, are classified into the low-risk group, and follow-up colonoscopy every 5–10 years is recommended for this group. In patients with hyperplastic polyps and those with only average risk, follow-up colonoscopy every 10 years is considered adequate.

Recently, in some studies, metabolic factors were shown to be associated with an increased prevalence of colorectal tumors. Limburg et al. and Elwing et al. demonstrated the association between diabetes mellitus and colorectal tumors [4, 5]. Otani et al. and Liu et al. showed a relationship between dyslipidemia and colorectal tumors [6, 7]. Kim et al. and Orranapalai et al. proved that metabolic syndrome was associated with colorectal tumors [8, 9]. The incidence rates of metabolic factors have dramatically increased in developed countries as a result of the high prevalence of obesity [10]. Therefore, metabolic factors rank as important risk factors for the increase of prevalence of colorectal tumors. Metabolic factors have also been suggested as risk factors for recurrence after endoscopic removal of colorectal adenomas. To the best of our knowledge, however, no study as yet has discussed whether the presence of metabolic factors might accelerate the development of recurrence after endoscopic removal of colorectal adenomas.

The aim of this study was to identify the risk factors for colorectal adenoma recurrence, focusing on metabolic factors, in addition to those adenoma-related factors that the AGA guidelines have established. We therefore constructed a scoring system with the identified risk factors for recurrence that could predict the recurrence rate according to the risk score.

## Methods

### Study participants

**Figure 1:**
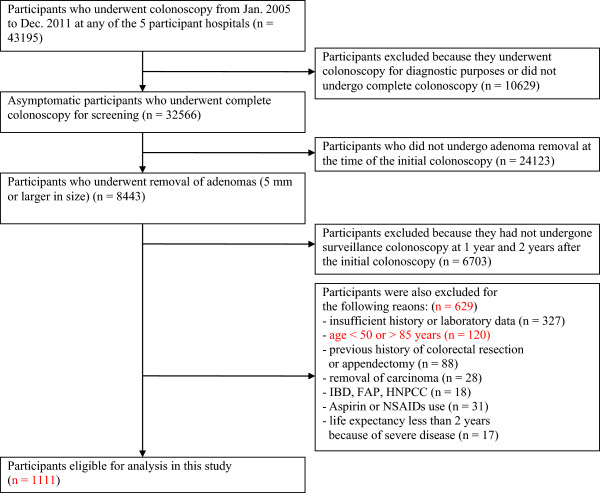
**Inclusion and exclusion criteria of the study participants.**

This multicenter retrospective study was conducted with the participation of 5 community hospitals in Japan, including Chigasaki Municipal Hospital, Yokohama Rousai Hospital, Tokyo Metropolitan Hiroo Hospital, Machida Municipal Hospital, and NTT Medical Center Tokyo. We analyzed the medical records of 43,195 patients who had undergone colonoscopy between January 2005 and December 2011 at any one of the 5 hospitals. Among these, only those who had undergone complete colonoscopy for the purpose of screening were enrolled in this study (n = 32,566); the remaining 10,629 patients were excluded either because they had undergone colonoscopy for diagnostic purposes, or did not undergo complete colonoscopy. Next, 24,123 subjects who had not undergone removal of adenomas ≥5 mm in size at the time of the screening colonoscopy were excluded, and the remaining 8443 subjects who had undergone removal of adenomas ≥5 mm in size were included in this study (smaller adenomas have been shown to be of minimal clinical significance [11]). Furthermore, from among the 8443 subjects, we excluded 6703 subjects who did not undergo surveillance colonoscopy at 1 year and 2 years after the initial colonoscopy (that is, all the eligible patients enrolled in this study had undergone colonoscopy 3 times; colonoscopy is very cheap in Japan, and annual surveillance colonoscopies are common). To be eligible for this study, the participants had to have undergone complete colonoscopy at all the three examinations. A complete colonoscopy was considered to include: colonoscopy up to the level of the cecum, good bowel preparation, and removal of all the detected adenomas. Thereafter, further participants were excluded for the following reasons (total n = 629): no history or no laboratory data available (n = 327); age less than 50 years old or greater than 85 years old (n = 120); history of colorectal resection or appendectomy (n = 88); history of removal of carcinoma (n = 28); history of inflammatory bowel disease (IBD), familial adenomatous polyposis (FAP) or hereditary non-polyposis colorectal cancer (HNPCC) (n = 18); history of regular use or use for over 7 days a month of aspirin or non-aspirin steroidal anti-inflammatory drugs (NSAIDs) (n =31); and patients with a life expectancy of less than 2 years because of severe diseases such as cancer, severe liver dysfunction, severe renal dysfunction, severe infection, etc. (n = 17); Finally, 1111 participants were included for the analysis in our study (Figure 1). To minimize the selection bias, all of the patients who had undergone colonoscopy at the 5 hospitals over the 7-year period were assessed, and all of the patients who fulfilled the inclusion criteria but not the exclusion criteria were enrolled in this study.

The study protocol was approved by the individual ethics committees of each of the 5 participating hospitals, that are Chigasaki Municipal Hospital, Yokohama Rousai Hospital, Tokyo Metropolitan Hiroo Hospital, Machida Municipal Hospital, and NTT Medical Center Tokyo.

### Data collection

A total of 20 data points at the initial colonoscopy and colorectal adenoma recurrence over the 2-year observation period were obtained from the medical records. The 20 items evaluated were: age at initial colonoscopy, gender, body mass index (BMI), current alcohol consumption, current cigarette smoking, the present history of hypertension, diabetes mellitus and dyslipidemia, fasting blood glucose (FBG), hemoglobin A1c (HbA1c), serum total cholesterol (TC), high-density lipoprotein cholesterol (HDL-C), low-density lipoprotein cholesterol (LDL-C) and triglycerides (TG), morphology/villous features/grade of dysplasia/location/size of the largest removed adenoma, and number of removed adenomas.

Siegel et al. showed that the prevalence of colorectal tumors increased in adults aged 65 and older [1]. So, age was categorized into ≥65 and <65. BMI was calculated as the weight (in kg) divided by height squared (in m^2^). Obesity was defined as BMI ≥25 [12]. BMI was classified into ≥25 and <25. Current alcohol intake history was defined as positive when the consumption was estimated to be in excess of 30 g/day. Present history of hypertension was defined as blood pressure ≥130/85 mmHg and/or current use of antihypertensive medication [13]. Present history of diabetes mellitus was diagnosed if the patients had a random glucose level of ≥200 mg/dl, FBG of ≥126 mg/dl, HbA1c of ≥6.5% or were taking antidiabetic medication [14]. Present history of dyslipidemia was defined as TG ≥150 mg/dl and/or HDL-C <40 mg/dl or were taking antidyslipidemic medication [13]. The FBG, HbA1c, TC, HDL-C, LDL-C, and TG were determined by laboratory testing at the time of the initial colonoscopy. FBG, HbA1c, HDL-C, and TG were classified into two groups with the above cutoff values. TC ≥220 and LDL ≥ 140 were adopted as cutoff values according to the Japanese definition [15]. For the largest among the removed adenomas at the time of the initial colonoscopy, we determined the presence/absence of >20% villous components, grade of dysplasia (high/low), location, and size. The location of the largest adenoma was classified as right-sided if it was in the cecum, ascending colon or transverse colon, and as left-sided if it was in the splenic flexure, descending colon, sigmoid colon or rectum. Colorectal adenoma recurrence was diagnosed if an adenoma(s) ≥5 mm in size was found in the surveillance colonoscopy, and both newly developed colorectal adenomas and colorectal adenomas recurring at the same location as that of the previously removed adenoma were included.

### Statistical analysis

Statistical analysis was conducted using the JMP 10.0 software. Chi-square analysis was used to compare differences in variables. Univariate analysis was performed by logistic regression analysis. Multivariate analysis was performed by multiple logistic regression analysis. The correlation between the risk score and colorectal adenoma recurrence were assessed by analysis of the receiver operating characteristic (ROC) curves. Differences were considered statistically significant at p <0.05.

## Results

### Baseline characteristics and recurrence of colorectal adenomas

The baseline clinical characteristics of the study participants with and without colorectal adenoma recurrence developing within 2 years of the initial colonoscopy are provided in Table 1 and clinical characteristics of the removed adenomas are shown in Table 2.

**Table 1:** **Clinical characteristics of the study participants (n = 1111)**

Parameters	Recurrence		***P***value
	Present (%)	Absent (%)	
Total	471 (42.4)	640 (57.6)	
Age (years)			0.808
**≥**65	275 (58.4)	369 (57.7)	
<65	196 (41.6)	271 (42.3)	
Gender			0.106
Male	370 (78.6)	476 (74.4)	
Female	101 (21.4)	164 (25.6)	
BMI (kg/m^2^)			<0.001
**≥**25	142 (30.1)	115 (18.0)	
<25	329 (69.9)	525 (82.0)	
Current alcohol consumption			0.407
Yes	265 (56.3)	376 (58.8)	
No	206 (43.7)	264 (41.3)	
Current cigarette smoking			0.011
Yes	174 (36.9)	190 (29.7)	
No	297 (63.1)	450 (70.3)	
Hypertension			0.019
Yes	280 (59.4)	335 (52.3)	
No	191 (40.6)	305 (47.7)	
Diabetes Mellitus			<0.001
Yes	149 (31.6)	129 (20.2)	
No	322 (68.4)	511 (79.8)	
Dyslipidemia			0.001
Yes	172 (36.5)	176 (27.5)	
No	299 (63.5)	464 (72.5)	
FBG (mg/dL)			<0.001
**≥**126	135 (28.7)	82 (12.8)	
<126	336 (71.3)	558 (87.2)	
HbA1c (%)			<0.001
**≥**6.5	109 (23.1)	71 (11.1)	
<6.5	362 (76.9)	569 (88.9)	
TC (mg/dL)			0.080
**≥**220	99 (21.0)	108 (16.9)	
<220	372 (79.0)	532 (83.1)	
HDL-C (mg/dL)			0.176
<40	56 (11.9)	60 (9.4)	
**≥**40	415 (88.1)	580 (90.6)	
LDL-C (mg/dL)			0.010
**≥**140	73 (15.5)	66 (10.3)	
<140	398 (84.5)	574 (89.7)	
TG (mg/dL)			<0.001
**≥**150	153 (32.5)	127 (19.8)	
<150	318 (67.5)	513 (80.2)

**Table 2:** **Clinical characteristics of the removed adenomas (n = 1111)**

Parameters	Recurrence		***P***value
	Present (%)	Absent (%)	
Total	471 (42.4)	640 (57.6)	
Morphology of the largest adenoma			<0.001
Non-polypoid lesion	79 (16.8)	48 (7.5)	
Polypoid lesion	392 (83.2)	592 (92.5)	
>20% Villous features of the largest adenoma			<0.001
Yes	178 (37.8)	145 (22.7)	
No	293 (62.2)	495 (77.3)	
Degree of differentiation of the largest adenoma			<0.001
high grade	199 (42.3)	114 (17.8)	
low grade	272 (57.7)	526 (82.2)	
Location of the largest adenoma			0.028
Right-sided colon	250 (53.1)	297 (46.4)	
Left-sided colon	221 (46.9)	343 (53.6)	
Diameter of the largest adenoma (mm)			<0.001
**≥**10	300 (63.7)	161 (25.2)	
<10	171 (36.3)	479 (74.8)	
Number of the removed adenomas			
**≥**3	320 (67.9)	121 (18.9)	<0.001
<3	151 (32.1)	519 (81.1)	

During the 2-year follow-up after the initial colonoscopy, 471 of the 1111 patients (42.4%) were identified as having recurrence of colorectal adenomas whereas the remaining 640 patients (57.6%) were free of recurrence (Table 1). Of the 471, the recurrence was identified at the 1-year surveillance colonoscopy in 313 patients (28.2%), and at the 2-year surveillance colonoscopy in the remaining 158 patients (14.2%).

The characteristics of recurrent adenomas are demonstrated in Table 3. Among recurrent adenomas, advanced adenomas occupied 10.8% (51/471). Location of the largest recurrent adenomas tended to exist in the right-sided colon.

**Table 3:** **Characteristics of recurrent adenomas (n = 471)**

Number of recurrent adenomas, median [interquartile range]	1 [1-2]
Diameter of the largest adenoma, median [interquartile range]	5 [5-8]
Appearance (%)	
Non-polypoid lesion	20 (4.2)
Polypoid lesion	451 (95.8)
Pathology (%)	
Advanced adenoma	51 (10.8)
Non-advanced adenoma	420 (89.2)
Location of the largest adenoma (%)	
Right-sided colon	290 (61.6)
Left-sided colon	191 (38.4)

### Univariate analysis to identify the risk factors for adenoma recurrence

Each of 20 risk factors evaluated was found to be statistically significant, except for the age, gender, current alcohol consumption, TC and HDL-C. Although older males were more likely to undergo adenoma removal at the time of the initial colonoscopy, age and gender were not identified as statistically significant factors influencing the risk of colorectal adenoma recurrence. While current alcohol consumption was not found to be statistically significant, current cigarette smoking was statistically significant. Metabolic factors such as the BMI, hypertension, diabetes mellitus and dyslipidemia were found to be statistically significant factors, as were the FBG, HbA1c, LDL-C and TG. Among the metabolic factors, TC and HDL-C were not statistically significant. In regard to the adenoma-related factors, non-polypoid lesion, presence of >20% villous components, presence of high-grade dysplasia, a larger maximum diameter and a larger number of removed adenomas were found to be statistically significant risk factors for colorectal adenoma recurrence. Right-sided adenomas were associated with a higher risk of recurrence than left-sided adenomas. The odds ratio and p value of each factor with univariate analysis are shown in Table 4.

**Table 4:** **Univariate analysis of risk factors for colorectal adenoma recurrence**

Factor	OR (95% CI)	***p***value
*Age ≥65	1.03 (0.81-1.31)	0.808
*Gender; Male	1.26 (0.95-1.67)	0.106
*BMI ≥25 kg/m^2^	1.97 (1.49-2.61)	<0.001
*Current alcohol consumption; Yes	0.90 (0.71-1.15)	0.407
*Current cigarette smoking; Yes	1.39 (1.08-1.79)	0.011
*Hypertension; Yes	1.33 (1.05-1.70)	0.019
Diabetes Mellitus; Yes	1.83 (1.39-2.41)	<0.001
Dyslipidemia; Yes	1.52 (1.17-1.96)	0.001
*FBG ≥126 mg/dl	2.73 (2.01-3.71)	<0.001
HbA1C ≥6.5 %	2.41 (1.74-3.35)	<0.001
TC ≥220 mg/dl	1.31 (0.97-1.78)	0.080
HDL-C <40 mg/dl	1.30 (0.89-1.92)	0.176
*LDL-C ≥140 mg/dl	1.60 (1.12-2.28)	0.010
*TG ≥150 mg/dl	1.94 (1.48-2.56)	<0.001
*Morphology of the largest adenoma; Non-polypoid lesion	2.49 (1.70-3.64)	<0.001
* > 20% Villous features of the largest adenoma; Yes	2.07 (1.59-2.70)	<0.001
*Degree of differentiation of the largest adenoma; high grade	3.38 (2.57-4.43)	<0.001
*Location of the largest adenoma; Right-sided colon	1.31 (1.03-1.66)	0.028
*Diameter of the largest adenoma ≥10 mm	5.22 (4.03-6.76)	<0.001
*Number of the removed adenomas ≥3	9.09 (6.89-11.99)	<0.001

*Risk factors that were used in multivariate analysis.

### Multivariate analysis to identify independent risk factors for adenoma recurrence

Multiple logistic regression analysis was performed with 15 of 20 risk factors. There was collinearity among a present history of diabetes mellitus, FBG and HbA1c. Among these 3 factors, FBG was used in the multivariate analysis because the odds ratios of FBG were maximum among these in the univariate analysis. There was also collinearity among a present history of dyslipidemia, TC, HDL-C, LDL-C and TG. The odds ratios of LDL-C and TG were greater than that of others, and the correlation coefficient between LDL-C and TG was low (correlation coefficient = 0.0992). So, among these factors, LDL-C and TG were used in the multivariate analysis.

The results from the multivariate analysis identified 7 factors, namely, age, BMI, FBG, dysplasia grade/location/size of the largest adenoma, and number of the removed adenomas as being statistically significant factors influencing the risk of colorectal adenoma recurrence. Villous features were almost statistically significant (p = 0.063). The odds ratio and p value of each factor from the multivariate analysis are shown in Table 5.

**Table 5:** **Multivariate analysis of risk factors for colorectal adenoma recurrence**

Factor	OR (95% CI)	***p***value
*Age ≥65	1.38 (1.01-1.89)	0.045
Gender; Male	1.00 (0.69-1.44)	0.999
*BMI ≥25 kg/m^2^	1.70 (1.19-2.44)	0.003
Current alcohol consumption;Yes	0.97 (0.69-1.36)	0.870
Current cigarette smoking; Yes	1.20 (1.08-1.79)	0.288
Hypertension; Yes	1.24 (0.91-1.68)	0.169
*FBG ≥126 mg/dl	2.04 (1.40-2.98)	<0.001
LDL-C ≥140 mg/dl	1.37 (0.86-2.15)	0.181
TG ≥150 mg/dl	1.29 (0.90-1.83)	0.159
Morphology of the largest adenoma; Non-polypoid lesion	1.06 (0.58-1.54)	0.825
* > 20% Villous features of the largest adenoma; Yes	1.56 (0.98-2.52)	0.063
*Degree of differentiation of the largest adenoma; high grade	2.40 (1.51-3.83)	<0.001
*Location of the largest adenoma; Right-sided colon	1.43 (1.06-1.94)	0.018
*Diameter of the largest adenoma ≥10 mm	2.89 (2.07-4.04)	<0.001
*Number of the removed adenomas ≥3	6.12 (4.52-8.34)	<0.001

*Risk factors that were adopted in our scoring system.

### Scoring system for calculating the recurrence rate of colorectal adenomas

Analysis was performed using 8 factors (Table 5): For adenoma-related factors, 5 factors, namely, villous features/grade of dysplasia/location/size of the largest adenoma, and number of the removed adenomas were included. For metabolic factors and other factors, 3 factors, namely, age, BMI, FBG were included. Seven of 8 factors were adopted because they were statistically significant in the multivariate analysis. Villous features were included because it was an established risk factor in the AGA guidelines, and was also nearly statistically significant in our study in the multivariate analysis including metabolic risk factors.

One point was scored for if each positive factor. But, because the odds ratio of the number of removed adenomas in the multivariate analysis was almost 3 times bigger than that of other factors, the number of removed adenomas counted as 3 points if it was positive. The risk score was calculated by summing up each individual score, and ranged from 0 to 10 points.

The correlation between the risk score (0–10 points) and colorectal adenoma recurrence during the 2-year follow-up after the initial colonoscopy was analyzed with a logistic regression analysis, which revealed a statistically significant correlation (p <0.001).

**Figure 2:**
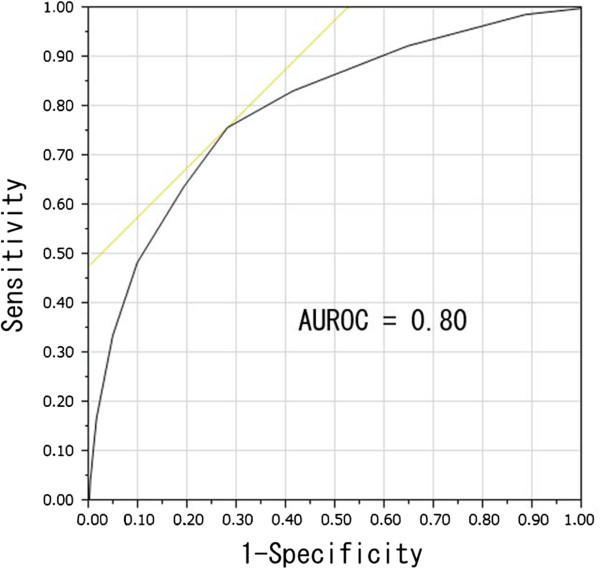
**ROC curve for the risk score and recurrence.** The AUROC for recurrence was determined to be 0.80 from ROC analysis.

ROC curve analysis revealed an AUROC for colorectal adenoma recurrence of 0.80 (Figure 2). The sensitivity, specificity, positive predictive value (PPV) and negative predictive value (NPV) for each risk score were calculated (Table 6). The recurrence rate of colorectal adenomas during the 2-year follow-up period after the initial colonoscopy, the number of cases showing adenoma recurrence and the total number of cases for each score are also shown in Table 6. When the cutoff value for the risk score was set at 3, the odds ratio of ≥3 points (3–10 points) relative to <3 points (0–2 points) for adenoma recurrence was determined to be 7.07 (95% CI 5.30–9.43). In those cases where the risk score was ≥3 (3–10) points, the colorectal adenoma recurrence rate during the 2-year period was 59.8%, and the sensitivity, specificity, PPV and NPV of the detection of adenoma recurrence were determined to be 83.2%, 58.8%, 59.8% and 82.6%, respectively (Table 7).

**Table 6:** **ROC analysis for the risk score and recurrence rate (n =1111)**

Risk score	Sensitivity (%)	Specificity (%)	PPV (%)	NPV (%)	Recurrence rate ^a^
0	100.0	0.0	42.4	100.0	7.5 (6/80)
1	98.7	11.6	45.1	92.5	16.4 (30/183)
2	92.4	35.5	51.3	86.3	22.4 (43/192)
3	83.2	58.8	59.8	82.6	29.2 (35/120)
4	75.8	72.0	66.6	80.2	50.0 (57/114)
5	63.7	80.9	71.1	75.2	54.6 (72/132)
6	48.4	90.3	78.6	70.4	68.6 (70/102)
7	33.6	95.3	84.0	66.1	79.0 (79/100)
8	16.8	98.6	89.8	61.7	88.1 (59/67)
9	4.3	99.8	95.2	58.6	93.8 (15/16)
10	1.1	100.0	100.0	57.9	100.0 (5/5)

^a^Recurrence rate during the 2-year period after the initial colonoscopy (%) (number of cases showing recurrence/total number of cases, for each risk score).

**Table 7:** **Cutoff value of the risk score for predicting recurrence (n = 1111)**

Risk score	Recurrence		Total
	Present	Absent	
0–2	79	376	455
3–10	392	264	656

The odds ratio was 7.07 (95% CI 5.30–9.43) (0–2 vs. 3–10).

The recurrence rate for the risk score 3–10 was 59.8% (392/656).

The sensitivity, specificity, PPV and NPV of a risk score 3–10 for the prediction of recurrence were 83.2%, 58.8%, 59.8% and 82.6%, respectively.

## Discussion

As suggested by the AGA guidelines, we confirmed with a multivariate analysis in this study that advanced adenomas or the presence of ≥3 adenomas were associated with an increased risk of colorectal adenoma recurrence [2] (note that villous features were nearly statistically significant). Furthermore, our multivariate analysis also revealed that age, 2 metabolic factors (elevated BMI, increased FBG) and adenomas in the right-sided colon at the initial colonoscopy were associated with an increased risk of colorectal adenoma recurrence.

Age and gender have been reported as risk factors for the prevalence of colorectal tumors [1]. Zauber et al. demonstrated that the presence of colorectal adenomas was higher in men and among elderly people [3]. However, age and sex still remain controversial from the point of view of the risk of recurrence of adenomas [2]. In our study, age was also identified as a risk factor of recurrence, while sex was not statistically significant for recurrence.

Among patients in whom colorectal adenomas were removed at the time of the initial colonoscopy, the estimated recurrence rate at surveillance colonoscopy performed within 3–4 years has been reported as 15–60% [16, 17]. In Japan, Yamaji et al. estimated a colorectal tumor recurrence rate of 7.2% per year in cases with no initial tumors, 19.3% per year in those with small adenomas, and 22.9% per year in those with advanced lesions [18]. In our study, the recurrence rate during a 2-year follow-up period after the initial screening colonoscopy was 42.4%, being largely consistent with previous reports.

At the time of surveillance colonoscopy, it is often difficult to differentiate between actual recurrence and detection of an adenoma missed in the earlier examination. On the other hand, from the clinical standpoint, strict differentiation between the two is not always necessary. Irrespective of whether the lesions identified on surveillance colonoscopy are recurrent or missed lesions, the lesions need to be removed. In our study, the recurrence rate at the 1-year surveillance colonoscopy was 28.2%, while that at the 2-year surveillance colonoscopy was 14.2%. This is possibly because of a substantial reduction of the miss rate resulting from the 2-year surveillance colonoscopy having been conducted after 2 earlier colonoscopies (initial and at 1 year). For the same reason, the recurrence rate at the 2-year surveillance colonoscopy (14.2%) is more likely to be closer to the actual recurrence rate per year. This rate is largely consistent with the rate reported by Yamaji et al. [18]. Furthermore, the difference in the recurrence rate between the 1-year and 2-year follow-up colonoscopies was 14.0% (=28.2%–14.2%), which may correspond to the miss rate of detection. Rex et al. reported that the miss rate for adenomas ≥10 mm in diameter was 6%, for adenomas 6–9 mm in diameter was 13%, and for adenomas ≤5 mm in diameter was 27% [19]. Pickhardt et al. reported from their virtual colonoscopy study that conventional colonoscopy failed to detect 12% of lesions ≥10 mm in diameter [20]. Our results were consistent with these miss rates.

The main pathophysiological abnormality associated with increased levels of metabolic factors is visceral fat deposition. Visceral fat deposition is known to be associated with insulin resistance, hyperinsulinemia and high levels of IGF I, which are thought to influence carcinogenic processes by increasing the cell proliferative activities and reducing apoptosis [8, 21]. Obesity-related inflammation and oxidative stress are also thought to increase the risk of development of colorectal tumors [22]. Visceral adipose tissue is considered to be an endocrine tissue, as it releases some inflammatory cytokines such as C-reactive protein, tumor necrosis factor, interleukin-6, and some adipocytokines such as leptin and adiponectin [23]. These cytokines and adipocytokines are considered to increase the risk of development, as well as the growth, of colorectal tumors. These may be the mechanisms underlying the reported increased prevalence of colorectal adenoma associated with metabolic factors. In this study, we demonstrated with the multivariate analysis that BMI was associated with the risk of recurrence of colorectal adenomas.

Diabetes mellitus is known to be associated with an increased prevalence of CRC [4], although its association with the prevalence of colorectal adenomas has not yet been sufficiently investigated. Nevertheless, among patients with obesity, a strong correlation has been reported between the presence of adenomas and diabetes mellitus [5]. Three factors, namely a present history of diabetes mellitus, FBG and HbA1c, had collinearity. In our study, FBG was used in the multivariate analysis, because its odds ratio was maximum in the univariate analysis among these 3 factors. FBG then remained as a statistically significant risk factor of colorectal adenoma recurrence in the multivariate analysis.

Some studies have reported that metabolic factors may be strongly associated with the presence of adenomas in the right-sided colon and multiple (three or more) adenomas [8, 24]. On the other hand, screening colonoscopy has been found to confer less protection against future development of CRC in the case of right-sided than left-sided adenomas [16, 25]. Poorer preparation and a more unfavorable anatomic configuration of the right-sided colon compromising the visibility of lesions are possible reasons for the higher miss rates of adenomas in the right-sided colon than in the left-sided colon [16]. In our study, location of the largest adenoma in the right-sided colon was also identified as a risk factor for recurrence. Therefore, endoscopists should pay particular attention to the right side of the colon, both at the time of the initial screening colonoscopy and at the time of the surveillance colonoscopy. It must be emphasized here that sigmoidoscopy is not adequate for surveillance after removal of adenomas.

Our study demonstrated that the higher the risk score, the higher the recurrence rate. ROC analysis revealed 3 as the optimal cutoff value as the risk score for predicting the risk of colorectal adenoma recurrence during the 2-year period from the initial colonoscopy, as it was associated with a very high AUROC and sensitivity. In those cases where the risk score was ≥3 points (3–10 points), the 2-year colorectal adenoma recurrence rate was calculated to be 59.8% (sensitivity 83.2%). Therefore, surveillance colonoscopy within 2 years may be a safe option for these patients.

Our study had some limitations. We were unable to obtain any waist circumference data. so it was impossible to analyze the association between the presence/absence of metabolic syndrome and colorectal adenoma recurrence.

Recently, colonoscopic quality indicators such as adenoma detection rates, withdrawal time and cecal intubation time are proposed. But these data couldn’t be obtained from medical records retrospectively. Each 5 hospitals conduct 2000–6000 colonoscopies per year, and all of colonoscopists in 5 hospitals were certified from Japan gastroenterological endoscopy society. Then, recurrence rate of adenoma of 5 hospitals ranged from 40.6% to 43.7% (p = 0.9766). There were no significant differences in recurrence rate between 5 hospitals. So, the bias according to the colonoscopists guess to be small.

Most of the participants of this study were followed up for only 2 years after the initial screening colonoscopy; and only a few patients were followed up for 3 years or more. Therefore, we could not collect the 3-year surveillance data.

A recent study has suggested that the use of aspirin or NSAIDs may be associated with a reduced prevalence of adenomas, and probably cancer [26]. However, among the participants who were eligible for analysis in this study, only 31 were taking aspirin/NSAIDs, and they were excluded from this study because the sample size was too small to allow reasonable analysis.

Family history of CRC has been reported as a risk factor for colorectal adenoma recurrence [2]. However, according to the medical records, the number of patients with a family history of CRC among the eligible participants was insufficient to allow reliable statistical analysis. Therefore, we did not include this factor in the analyses in our study.

Further prospective studies using larger sample sizes may be warranted to determine the risk of colorectal adenoma recurrence at 3 years or later after screening colonoscopy, as well as that associated with the above factors.

## Conclusions

In addition to the risk factors included in the AGA guidelines, we demonstrated that age, BMI, FBG and adenomas located in the right-sided colon were associated with an increased risk of development of recurrent colorectal adenomas after removal of adenomas at the initial colonoscopy. Based on this finding, we then constructed a scoring system to predict the recurrence rate according to our risk score.
